# Rezivertinib in *EGFR*-Mutated Non-Small Cell Lung Cancer Patients with Central Nervous System Metastasis: Central Nervous System Efficacy from the Phase III REZOR Study

**DOI:** 10.34133/cancomm.0018

**Published:** 2026-03-31

**Authors:** Sheng Yang, Yanqiu Zhao, Meili Sun, Minghong Bi, Bo Zhu, Zhaohong Chen, Huiqing Yu, Liangming Zhang, Lin Wu, Rui Zhou, Wenxiu Yao, Xingya Li, Zhigang Han, Ke Wang, Lijun Wang, Meiling Wen, Yanzhen Guo, Yingcheng Lin, Shenghua Sun, Shuliang Guo, Tienan Yi, Wenhua Zhao, Zhuang Yu, Jianwen Qin, Yueyin Pan, Zhiyong He, Feng Ye, Huaqiu Shi, Jian Fang, Rui Ma, Hong Lu, Hua Zhang, Jianhua Shi, Jinghua Gao, Jiuwei Cui, Manxiang Li, Shanyong Yi, Shundong Cang, Yongqian Shu, Don Zhang, Jirong Peng, Feng Gao, Tingting Wang, Anqi Zhou, Yuankai Shi

**Affiliations:** ^1^ Department of Medical Oncology, Beijing Key Laboratory of Key Technologies for Early Clinical Trial Evaluation of Innovative Drugs for Major Diseases, National Cancer Center/National Clinical Research Center for Cancer/Cancer Hospital, Chinese Academy of Medical Sciences & Peking Union Medical College, Beijing, P. R. China.; ^2^ Respiratory Department of Internal Medicine, The Affiliated Cancer Hospital of Zhengzhou University & Henan Cancer Hospital, Zhengzhou, Henan, P. R. China.; ^3^ Department of Oncology, Central Hospital Affiliated to Shandong First Medical University, Jinan, Shandong, P. R. China.; ^4^ Department of Medical Oncology, The First Affiliated Hospital of Bengbu Medical University, Bengbu, Anhui, P. R. China.; ^5^ Department of Oncology, Institute of Cancer, Xinqiao Hospital, Third Military Medical University, Chongqing, P. R. China.; ^6^ Department of Oncology, People’s Hospital of Deyang City, Deyang, Sichuan, P. R. China.; ^7^ Department of Geriatric Oncology, Chongqing University Cancer Hospital, Chongqing, P. R. China.; ^8^ Department of Medical Oncology, Yantai Yuhuangding Hospital, Yantai, Shandong, P. R. China.; ^9^ Department of Thoracic Medical Oncology, Hunan Cancer Hospital/The Affiliated Cancer Hospital of Xiangya School of Medicine, Central South University, Changsha, Hunan, P. R. China.; ^10^ Department of Respiratory and Critical Care Medicine, The Second Xiangya Hospital of Central South University, Changsha, Hunan, P. R. China.; ^11^ Department of Medical Oncology, Sichuan Cancer Hospital/Cancer Hospital Affiliated to University of Electronic Science and Technology of China, Chengdu, Sichuan, P. R. China.; ^12^ Department of Oncology, The First Affiliated Hospital of Zhengzhou University, Zhengzhou, Henan, P. R. China.; ^13^ First Department of Medicine, Affiliated Cancer Hospital of Xinjiang Medical University, Urumqi, Xinjiang, P. R. China; ^14^ Department of Respiratory and Critical Care Medicine, West China Hospital of Sichuan University, Chengdu, Sichuan, P. R. China.; ^15^ Cancer Center, The Second Affiliated Hospital of Xingtai Medical College, Xingtai, Hebei, P. R. China.; ^16^ Department of Medical Oncology, The First Affiliated Hospital of University of South China, Hengyang, Hunan, P. R. China; ^17^ Department of Medical Oncology, The First Affiliated Hospital of Henan University of Science & Technology, Luoyang, Henan, P. R. China; ^18^ Department of Medical Oncology, Cancer Hospital of Shantou University Medical College, Shantou, Guangdong, P. R. China.; ^19^ Department of Pulmonary and Critical Care Medicine, Third Xiangya Hospital of Central South University, Changsha, Hunan, P. R. China.; ^20^ Department of Respiratory and Critical Care Medicine, The First Affiliated Hospital of Chongqing Medical University, Chongqing, P. R. China; ^21^ Department of Medical Oncology, Xiangyang Central Hospital, Xiangyang, Hunan, P. R. China.; ^22^ Department of Internal Medicine for Lung Cancer, Guangxi Medical University Cancer Hospital, Nanning, Guangxi, P. R. China.; ^23^ Department of Medical Oncology, The Affiliated Hospital of Qingdao University, Qingdao, Shandong, P. R. China.; ^24^ Department of Respiratory and Critical Medicine, Chest Hospital, Tianjin University, Tianjin, P. R. China.; ^25^ Department of Oncology, The First Affiliated Hospital of University of Science and Technology of China, Division of Life Sciences and Medicine, University of Science and Technology of China, Hefei, Anhui, P. R. China.; ^26^ Department of Medical Oncology, Clinical Oncology School of Fujian Medical University, Fujian Cancer Hospital, National Health Commission (NHC) Key Laboratory of Cancer Metabolism, Fuzhou, Fujian, P. R. China.; ^27^ Department of Medical Oncology, Xiamen Key Laboratory of Antitumor Drug Transformation Research, The First Affiliated Hospital of Xiamen University, School of Medicine, Xiamen University, Xiamen, Fujian, P. R. China.; ^28^ Department of Medical Oncology, First Affiliated Hospital of Gannan Medical University, Ganzhou, Jiangxi, P. R. China.; ^29^ Department of Thoracic Oncology, Beijing Cancer Hospital, Beijing, P. R. China.; ^30^ Department of Thoracic Oncology, Liaoning Cancer Hospital & Institute, Shenyang, Liaoning, P. R. China.; ^31^ Department of Oncology, Huaihe Hospital of Henan University, Kaifeng, Henan, P. R. China.; ^32^ Department of Medical Oncology, The First Affiliated Hospital of Xinjiang Medical University, Urumqi, Xinjiang, P. R. China.; ^33^ Department of Medical Oncology, Linyi Cancer Hospital, Linyi, Shandong, P. R. China.; ^34^ Department of Medical Oncology, Cangzhou Central Hospital, Cangzhou, Hebei, P. R. China.; ^35^ Oncology Center, Oncology Department, First Hospital of Jilin University, Changchun, Jilin, P. R. China.; ^36^ Department of Respiratory and Critical Care Medicine, The First Affiliated Hospital of Xi’an Jiaotong University, Xi’an, Shaanxi, P. R. China.; ^37^ Department of Medical Oncology, Zhengzhou Central Hospital Affiliated to Zhengzhou University, Zhengzhou, Henan, P. R. China.; ^38^ Department of Oncology, Henan Provincial People’s Hospital, Zhengzhou, Henan, P. R. China.; ^39^ Department of Oncology, The First Affiliated Hospital of Nanjing Medical University (Jiangsu Province Hospital), Nanjing, Jiangsu, P. R. China.; ^40^ Department of Drug Discovery, Beta Pharma Inc., Princeton, NJ, USA.; ^41^ Department of Clinical Development, Beta Pharma (Shanghai) Co., Ltd., Shanghai, P. R. China.

## Abstract

**Background:** From 2019 July 15 to 2022 February 14, the REZOR study enrolled 369 treatment-naïve patients with locally advanced or metastatic non-small cell lung cancer harboring *EGFR* mutations (exon 19 deletion or L858R mutation). Patients were randomly assigned 1:1 to receive either rezivertinib (180 mg/d) plus gefitinib placebo or gefitinib (250 mg/d) plus rezivertinib placebo. Previous results demonstrated significantly improved progression-free survival (PFS) with rezivertinib versus gefitinib and a favorable safety profile. Here, we update the analyses of central nervous system (CNS) outcomes in patients with baseline CNS metastases. **Methods:** All patients underwent brain magnetic resonance imaging at baseline and each subsequent efficacy evaluation until radiological disease progression or any other treatment discontinuation criteria were met. *EGFR* mutation status was determined by testing using tissue or plasma samples during screening. Patients with stable, asymptomatic CNS metastasis were eligible for enrollment. The CNS full analysis set (cFAS) comprised patients with baseline CNS metastasis identified on magnetic resonance imaging and evaluated by blinded independent central review according to the Response Assessment in Neuro-Oncology Brain Metastases criteria. Patients with measurable CNS target lesions formed the CNS evaluable-for-response set (cEFR). **Results:** As of the 2023 November 30 data cutoff, 159 patients had baseline CNS metastasis in the cFAS (rezivertinib: *n* = 81; gefitinib: *n* = 78) and 25 in the cEFR (rezivertinib: *n* = 12; gefitinib: *n* = 13) per blinded independent central review. In the cFAS, 59 CNS PFS events occurred (rezivertinib: *n* = 30; gefitinib: *n* = 29). Median CNS PFS was significantly longer with rezivertinib (24.9 months; 95% confidence interval [CI], 16.5 months-not estimable [NE]) than with gefitinib (15.2 months; 95% CI, 10.5 months-NE), with a hazard ratio of 0.58 (95% CI, 0.34 to 0.99; *P* = 0.047). In the cEFR, the CNS objective response rate was 83.3% (95% CI, 51.6% to 97.9%) with rezivertinib and 76.9% (95% CI, 46.2% to 95.0%) with gefitinib (odds ratio = 1.50; 95% CI, 0.20 to 11.0; *P* = 0.690). No new safety findings were observed. **Conclusions:** Rezivertinib demonstrated a statistically significant superior CNS efficacy over gefitinib as first-line treatment in advanced *EGFR*-mutated non-small cell lung cancer patients with baseline CNS metastases. The safety profile was consistent with previous analyses. **Trial registration:** NCT03866499 (ClinicalTrials.gov).

## Background

Lung cancer has the highest incidence and mortality among all malignancies, of which the adenocarcinoma was the most common subtype among East Asian populations [1]. Approximately 80% to 85% of lung cancers are classified as non-small cell lung cancer (NSCLC), and over 40% of lung adenocarcinoma in East Asian patients harbor epidermal growth factor receptor (*EGFR*) mutations [2–6]. Currently, EGFR tyrosine kinase inhibitors (TKIs) are the standard first-line treatment for patients with *EGFR* mutations, with studies showing that third-generation EGFR TKIs have superior efficacy compared with first-generation EGFR TKIs in the first-line setting [7–14]. Up to 50% of NSCLC patients with *EGFR*-sensitizing mutations have been reported to develop central nervous system (CNS) metastases, which are associated with poor prognosis [15]. In addition to stereotactic radiosurgery and whole-brain radiation therapy, EGFR TKIs have emerged as an important systemic treatment option for patients with CNS metastases in the first-line setting. Compared with first- and second-generation EGFR TKIs, which surpassed conventional chemotherapy but have restricted CNS efficacy, third-generation EGFR TKIs have demonstrated substantially improved CNS outcomes as monotherapy [15–20]. For patients with CNS metastasis harboring the *EGFR* T790M mutation, osimertinib [16,17], aumolertinib [21], furmonertinib [22–24], rezivertinib [25–27], and limertinib [28] also demonstrated promising efficacy. Furthermore, combination regimens have shown superior CNS progression-free survival (PFS) over single-agent therapy, as demonstrated by osimertinib plus chemotherapy in the FLAURA2 trial [29] and amivantamab plus lazertinib in the MARIPOSA trial [30]. Therefore, EGFR TKIs have been the standard treatment of NSCLC patients with CNS metastasis harboring *EGFR*-sensitizing mutations or *EGFR* T790M mutation [31–34].

Rezivertinib (BPI-7711) is a novel, irreversible third-generation EGFR TKI codeveloped by Beta Pharma (Shanghai) Co., Ltd. (Shanghai, China) and Beta Pharma Inc. (Princeton, New Jersey, USA). It was designed with structural innovation, incorporating an N,N-dimethyl-substituted oxyethylamine side chain to balance lipophilicity and hydrophilicity. This specific design helps prevent efflux by P-glycoprotein or breast cancer resistance protein, thereby enabling effective blood–brain barrier penetration [13]. Prior clinical studies demonstrated promising efficacy and a favorable safety profile when treating NSCLC patients with CNS metastasis and *EGFR* T790M mutation who had previously received the treatment of first- or second-generation EGFR TKIs [25–27]. The REZOR phase III study previously showed that rezivertinib significantly prolonged PFS and offered a favorable safety profile compared to gefitinib in the first-line setting [13]. In the present analysis, we report updated CNS outcomes from the REZOR study of rezivertinib versus gefitinib in patients with baseline CNS metastases.

## Materials and Methods

### Study design and treatment

The REZOR was a multicenter, randomized phase III study (NCT03866499), conducted at 50 hospitals in China (Table S1). Eligible patients were stratified by *EGFR* mutation type (exon 19 deletion or L858R mutation) and the presence of CNS metastasis at baseline. They were then randomly assigned (1:1) to each treatment group, to receive either oral rezivertinib (180 mg/d) plus gefitinib placebo or oral gefitinib (250 mg/d) plus rezivertinib placebo. While treatment was discontinued upon unacceptable toxicity, disease progression, or other treatment discontinuation criteria, investigators could allow treatment beyond progression for clinical benefit. Each treatment cycle lasted for 21 d. Eligible patients in the gefitinib group who progressed and were confirmed to have a secondary *EGFR* T790M mutation could cross over to receive open-label rezivertinib treatment until predefined discontinuation criteria were met. A masking maintenance plan was executed to ensure blinding remained intact throughout the study. This included randomization, drug coding, dosing, data monitoring, data management, and statistical analysis, lasting until the protocol’s predefined unmasking conditions were satisfied. Details of the REZOR study can be found in the previous publication [13].

### Participants

The REZOR study enrolled patients ≥18 years old with histologically or cytologically confirmed NSCLC and an Eastern Cooperative Oncology Group performance status of 0 to 1. All patients were treatment-naïve, had locally advanced or metastatic NSCLC, and were deemed unsuitable for radical surgery or radiotherapy. They were required to have ≥1 measurable lesion per the Response Evaluation Criteria in Solid Tumors (RECIST) version 1.1. Central laboratory testing using the Cobas *EGFR* mutation test (version 2, Roche Diagnostics, South Branchburg, NJ) on tissue and/or plasma samples was required to confirm the presence of targetable *EGFR* mutations (exon 19 deletion or L858R mutation). The Cobas DNA Sample Preparation Kit (for formalin-fixed, paraffin-embedded tissue; Roche Diagnostics; catalog no. 05985536190) and the Cobas cfDNA Sample Preparation Kit (for plasma; Roche Diagnostics; P/N: 07247737190) were used for manual extraction of nucleic acids. Exon 20 insertions were excluded. Patients were required to receive brain scans with plain plus contrast-enhanced magnetic resonance imaging (MRI) at screening, and those with asymptomatic CNS metastasis were allowed entry if they had abstained from steroids and/or antiepileptic drugs for at least 7 d before the first dose.

### Ethics

The study complied with the Declaration of Helsinki, the International Council on Harmonization Good Clinical Practice Guidelines, and relevant regulatory requirements. Each participating hospital’s institutional review board or independent ethics committee approved the protocol (the approval number of the ethics committee of the leading hospital, National Cancer Center/Cancer Hospital, Chinese Academy of Medical Sciences & Peking Union Medical College, was 19-039/1824). Informed consent was obtained from every patient before enrollment.

### Assessments

For patients with brain metastases at baseline, the assessments were conducted during the screening and at each subsequent efficacy evaluation. Within 18 months (78 weeks) after randomization, follow-up assessments should be conducted every 6 weeks (2 cycles), up to cycle 25, day 1 (C25D1), and then every 12 weeks (4 cycles) from C27D1 onward until radiological disease progression. The primary end point of the REZOR study was PFS evaluated by blinded independent central review (BICR) per the RECIST version 1.1. In this analysis, the efficacy for patients with CNS metastasis was measured by BICR according to the Response Assessment in Neuro-Oncology criteria for Brain Metastases (RANO-BM) [35]. CNS efficacy end points included CNS objective response rate (ORR), CNS disease control rate (DCR), CNS duration of response (DoR), and CNS PFS. The full analysis set (FAS) involved all randomized patients from the REZOR study. The CNS full analysis set (cFAS) included patients with measurable or nonmeasurable CNS lesions at baseline. In contrast, the CNS evaluable-for-response set (cEFR) was restricted to those patients who had measurable CNS lesions at baseline. Safety was assessed by investigators according to the National Cancer Institute Common Terminology Criteria for Adverse Events version 4.03.

### Statistical analysis

In this analysis, the median CNS DoR and CNS PFS, along with their 95% confidence interval (CI), were calculated by the Kaplan–Meier method. The hazard ratios (HRs) for rezivertinib versus gefitinib and corresponding 95% CI were estimated using a stratified Cox proportional hazards model, and *P* values were calculated using the log-rank test. The calculation of both the CNS ORR and the CNS DCR was based on the best overall response (BOR) achieved during the study. The 95% CIs for these rates were established via the Clopper–Pearson method. The cumulative incidence of the first event being CNS progression (prior to non-CNS progression or death) was estimated using a cause-specific hazard model within a competing risk analysis. Statistical analyses were performed with SAS version 9.4.

CNS ORR represented the percentage of patients with CNS metastases at baseline who achieved a complete response (CR) or partial response (PR). CNS DCR included patients with CNS metastases at baseline who achieved CR, PR, or stable disease that persisted for at least 39 d after initiating treatment. For patients who achieved CNS response (CR or PR), CNS DoR was calculated as the interval from the first CNS CR or PR until the occurrence of either CNS disease progression or death from any cause. CNS PFS was defined as the time relapsed from randomization until the event of CNS disease progression or death.

## Results

### Participants

**Fig. 1.**
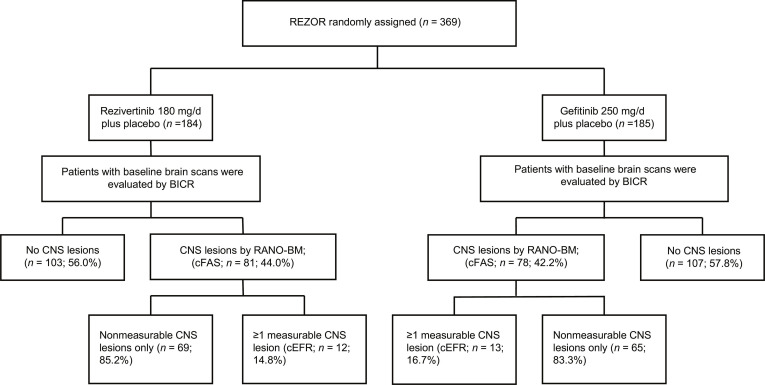
Flowchart of this study. BICR, blinded independent central review; cFAS, CNS full analysis set; CNS, central nervous system; cEFR, CNS evaluable for response set; FAS, full analysis set; MRI, magnetic resonance imaging; RANO-BM: Response Assessment in Neuro-Oncology criteria for Brain Metastases.

The REZOR study screened a total of 695 patients across 50 hospitals in China between 2019 July 15 and 2022 February 14. Ultimately, 369 eligible patients were randomized (1:1 ratio) to receive either rezivertinib (180 mg/d) orally plus gefitinib placebo orally (*n* = 184) or gefitinib (250 mg/d) orally plus rezivertinib placebo orally (*n* = 185). By the data cutoff date on 2023 November 30, 159 (rezivertinib: *n* = 81; gefitinib: *n* = 78) patients with baseline CNS metastasis were included in cFAS, and 25 (rezivertinib: *n* = 12; gefitinib: *n* = 13) were included in cEFR assessed by BICR according to RANO-BM (Fig. 1). Patient baseline characteristics of the cFAS and FAS were balanced between the 2 groups (Table 1). The median CNS target lesion size at baseline was comparable in the 2 groups (15.2 mm [range: 10.2 to 67.5 mm] in the rezivertinib group and 17.6 mm [range: 11.5 to 54.7 mm] in the gefitinib group). Before enrollment, 2 patients from the rezivertinib group and no patients from the gefitinib group underwent CNS radiotherapy due to CNS metastasis.

**Table 1.** Baseline demographic and clinical characteristics of patients in the FAS and the cFAS

Characteristics	FAS		cFAS	
	Rezivertinib (*n* = 184)	Gefitinib (*n* = 185)	Rezivertinib (*n* = 81)	Gefitinib (*n* = 78)
Age, years				
Median (range)	61.0 (32.0–86.0)	62.0 (30.0–81.0)	59.0 (32.0–79.0)	60.5 (35.0–79.0)
<65, *n* (%)	113 (61.4)	104 (56.2)	52 (64.2)	46 (59.0)
≥65, *n* (%)	71 (38.6)	81 (43.8)	29 (35.8)	32 (41.0)
Sex, *n* (%)				
Male	72 (39.1)	80 (43.2)	34 (42.0)	31 (39.7)
Female	112 (60.9)	105 (6.8)	47 (58.0)	47 (60.3)
ECOG PS, *n* (%)				
0	41 (22.3)	32 (17.3)	13 (16.0)	8 (10.3)
1	143 (77.7)	153 (82.7)	68 (84.0)	70 (89.7)
*EGFR* mutation assessment sample type ^a^, *n* (%)				
Tissue	130 (70.7)	140 (75.7)	57 (70.4)	59 (75.6)
Plasma	75 (40.8)	61 (33.0)	32 (39.5)	26 (33.3)
*EGFR* mutation subtype, *n* (%)				
Exon 19 deletion	93 (50.5)	97 (52.4)	37 (45.7)	39 (50.0)
L858R mutation	91 (49.5)	88 (47.6)	44 (54.3)	39 (50.0)
Median CNS target lesion size (range), mm	15.2 (10.2–67.5)	17.6 (11.5–54.7)	15.2 (10.2–67.5)	17.6 (11.5–54.7)
CNS radiotherapy history	2 (1.1)	0 (0.0)	2 (2.5)	0 (0.0)

^a^ Tissue and plasma samples were obtained from the same individuals and thus counted more than once. cFAS, CNS full analysis set; CNS, central nervous system; ECOG, Eastern Cooperative Oncology Group; PS, performance score; EGFR, epidermal growth factor receptor; FAS, full analysis set.

### Efficacy

**Fig. 2.**
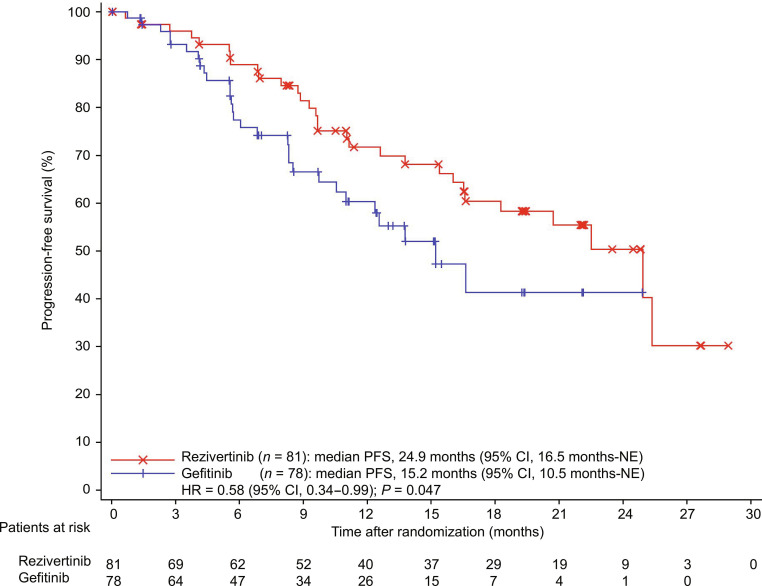
Kaplan–Meier curves of CNS PFS in cFAS assessed by BICR (*n* = 159). BICR, blinded independent central review; cFAS, CNS full analysis set; CI, confidence interval; CNS, central nervous system; FAS, full analysis set; HR, hazard ratio; PFS, progression-free survival; NE, not estimable.

As of the data cutoff date, the median follow-up time for CNS PFS in the cFAS was 19.3 months (95% CI, 16.6 to 22.1) in the rezivertinib group and 12.4 months (95% CI, 9.7 to 15.1) in the gefitinib group. In the cFAS, 59 (rezivertinib: *n* = 30; gefitinib: *n* = 29) CNS PFS events occurred, and the median CNS PFS was significantly prolonged in the rezivertinib group (24.9 months [95% CI, 16.5 months-not estimable (NE)]) compared with the gefitinib group (15.2 months [95% CI, 10.5 months-NE]), with an HR of 0.58 (95% CI, 0.34 to 0.99; *P* = 0.047; Table 2 and Fig. 2). Estimated CNS PFS rates at 6, 12, and 18 months were 88.9% (95% CI, 79.1% to 94.3%), 71.7% (95% CI, 59.2% to 81.0%), and 60.4% (95% CI, 46.9% to 71.5%) for the rezivertinib group, respectively, versus 77.4% (95% CI, 65.3% to 85.8%), 60.3% (95% CI, 46.4% to 71.7%), and 41.3% (95% CI, 24.1% to 57.8%) for the gefitinib group. The median CNS DoR was NE (95% CI, 19.3 months-NE) in the rezivertinib group and NE (95% CI, 7.0 months-NE) in the gefitinib group. Meanwhile, the CNS CR rate was 25.9% in the rezivertinib group versus 12.8% in the gefitinib group (Table 2). In cFAS, 10 patients had leptomeningeal nontarget lesions at baseline (each group with 5 patients, evaluated by BICR through MRI). All these patients showed BOR with non-CR/non-progressive disease (PD) for the leptomeningeal lesions. Among these patients, 2 from each group were also included in the cEFR with measurable CNS target lesions and achieved a BOR of CNS PR. Patients treated with rezivertinib exhibited a numerically longer CNS PFS compared with the gefitinib group (Table S2). No patients were confirmed to have developed new leptomeningeal lesions in both groups.

**Table 2.** CNS efficacy in the cEFR and the cFAS assessed by BICR

CNS efficacy	cFAS (*n* = 159)		cEFR (*n* = 25)	
	Rezivertinib (*n* = 81)	Gefitinib (*n* = 78) ^a^	Rezivertinib (*n* = 12)	Gefitinib (*n* = 13)
Median CNS PFS follow-up (95% CI), months	19.3 (16.6–22.1)	12.4 (9.7–15.1)	22.0 (16.6–23.5)	15.2 (6.9–22.1)
Median CNS PFS (95% CI), months	24.9 (16.5-NE)	15.2 (10.5-NE)	NC (8.0-NE)	12.6 (5.6-NE)
HR (95% CI)	0.58 (0.34–0.99)		0.30 (0.06–1.47)	
*P* value	0.047		0.138	
Estimated CNS PFS rates (95% CI), %				
6-month	88.9 (79.1–94.3)	77.4 (65.3–85.8)	91.7 (53.9–98.8)	76.9 (44.2–91.9)
12-month	71.7 (59.2–81.0)	60.3 (46.4–71.7)	73.3 (37.9– 90.6)	51.9 (22.5–75.0)
18-month	60.4 (46.9–71.5)	41.3 (24.1–57.8)	73.3 (37.9–90.6)	41.5 (14.4–67.2)
Median CNS DoR (95% CI), months	NE (19.3-NE)	NE (7.0-NE)	NE (6.6-NE)	NE (4.7-NE)
Best overall response, *n* (%)				
CR	21 (25.9)	10 (12.8)	0 (0.0)	0 (0.0)
PR	10 (12.3)	10 (12.8)	10 (83.3)	10 (76.9)
SD	1 (1.2)	1 (1.3)	1 (8.3)	1 (7.7)
Non-CR/Non-PD	43 (53.1)	52 (66.7)	0 (0.0)	0 (0.0)
PD	1 (1.2)	2 (2.6)	1 (8.3)	1 (7.7)
NE	5 (6.2)	3 (3.8)	0 (0.0)	1 (7.7)
CNS ORR, *n* (%)	31 (38.3)	20 (25.6)	10 (83.3)	10 (76.9)
95% CI, %	27.7–49.7	16.4–36.8	51.6–97.9	46.2–95.0
CNS DCR, *n* (%)	32 (39.5)	21 (26.9)	11 (91.7)	11 (84.6)
95% CI, %	28.8–51.0	17.5–8.2	61.5–99.8	54.6–98.1

^a^ One patient from the gefitinib group had no postbaseline CNS response assessment results.

BICR, blinded independent central review; cFAS, CNS full analysis set; cEFR, CNS evaluable for response set; CI, confidence interval; CNS, central nervous system; CR, complete response; DCR, disease control rate; FAS, full analysis set; HR, hazard ratio; ORR, objective response rate; PR, partial response; PD, progressive disease; NE, not estimable; PFS, progression-free survival; DoR, duration of response; SD, stable disease.

**Fig. 3.**
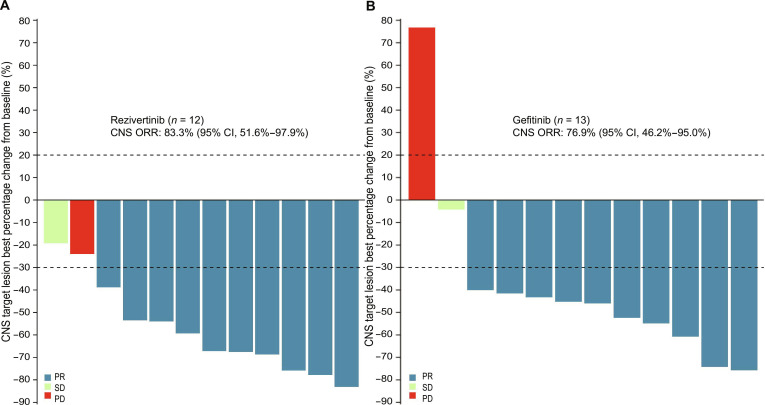
Best percentage changes in target lesions from baseline in cEFR assessed by BICR. (A) Best percentage changes in target lesions from baseline in cEFR assessed by BICR in the rezivertinib group. (B) Best percentage changes in target lesions from baseline in cEFR assessed by BICR in the gefitinib group. Note: The dashed line at 20% represents the boundary for the determination of PD, and the dashed line at −30% represents the boundary for the determination of PR. BICR, blinded independent central review; cEFR, CNS evaluable for response set; CI, confidence interval; CNS, central nervous system; ORR, objective response rate; PR, partial response; PD, progressive disease; SD, stable disease.

In the cEFR, the median follow-up time for CNS PFS in cEFR was 22.0 months (95% CI, 16.6 to 23.5) in the rezivertinib group and 15.2 months (95% CI, 6.9 to 22.1) in the gefitinib group. In total, 83.3% (10/12) of the patients in the rezivertinib group and 76.9% (10/13) in the gefitinib group achieved CNS PR. One patient in each group exhibited CNS PD as the BOR, that is, 8.3% (1/12) in the rezivertinib group and 7.7% (1/13) in the gefitinib group (Table 2). The CNS ORR was 83.3% (95% CI, 51.6% to 97.9%) for the rezivertinib group and 76.9% (95% CI, 46.2% to 95.0%) for the gefitinib group (Table 2). The CNS DCR was 91.7% (95% CI, 61.5% to 99.8%) for the rezivertinib group and 84.6% (95% CI, 54.6% to 98.1%) for the gefitinib group (Table 2). Estimated CNS PFS rates at 6, 12, and 18 months were 91.7% (95% CI, 53.9% to 98.8%), 73.3% (95% CI, 37.9% to 90.6%), and 73.3% (95% CI, 37.9% to 90.6%) for the rezivertinib group, respectively, versus 76.9% (95% CI, 44.2% to 91.9%), 51.9% (95% CI, 22.5% to 75.0%), and 41.5% (95% CI, 14.4% to 67.2%) for the gefitinib group. The median CNS DoR was NE (95% CI, 6.6 months-NE) in the rezivertinib group and NE (95% CI, 4.7 months-NE) in the gefitinib group (Table 2). The best percentage changes in target lesions from baseline in cEFR assessed by BICR are detailed in Table S3 and Fig. 3. Among the cEFR populations, the rezivertinib group showed a greater reduction in median best percentage change in target lesions from baseline over the gefitinib group [−63.2% (range: −83.1% to −19.1%) versus −45.6% (range: −75.7% to 76.7%); *P* = 0.157]. Meanwhile, the proportion of patients with over 50% reduction in target lesions from baseline in the rezivertinib group was 75.0% (9/12), while that in the gefitinib group was 38.5% (5/13; *P* = 0.098).

**Fig. 4.**
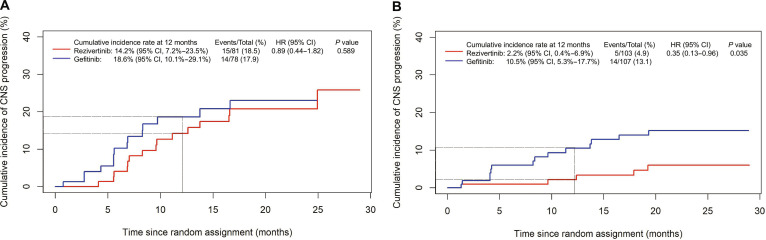
Cumulative incidence of CNS progression in patients (A) with CNS metastasis at baseline and (B) without CNS metastasis at baseline. CI, confidence interval; CNS, central nervous system; HR, hazard ratio.

For patients with baseline CNS metastases, the estimated probability of CNS progression at 6 months was 4.1% (95% CI, 1.1% to 10.5%) for the rezivertinib group and 10.2% (95% CI, 4.4% to 18.9%) for the gefitinib group, and at 12 months with 14.2% (95% CI, 7.2% to 23.5%) versus 18.6% (95% CI, 10.1% to 29.1%), respectively (HR = 0.89; 95% CI, 0.44 to 1.82; *P* = 0.589; Fig. 4A and Table S4). For patients without baseline CNS metastasis, the estimated 6-month probability of CNS progression was 1.0% (95% CI, 0.1% to 4.8%) for the rezivertinib group and 6.0% (95% CI, 2.5% to 11.9%) for the gefitinib group, while the 12-month probabilities were 2.2% (95% CI, 0.4% to 6.9%) and 10.5% (95% CI, 5.3% to 17.7%), respectively (HR = 0.35; 95% CI, 0.13 to 0.96, *P* = 0.035; Fig. 4B and Table S4). By the data cutoff date, the probability and time to develop new CNS lesions were calculated among patients with and without baseline CNS metastasis (Table 3). Among patients without baseline CNS metastasis, those in the rezivertinib group had a lower probability of developing new CNS lesions compared to those in the gefitinib group (4.9% versus 13.1%). Meanwhile, compared to the gefitinib group, the rezivertinib group showed a prolonged time to the development of new CNS lesions among patients with or without baseline CNS metastasis. The median time to the development of new CNS lesions was 9.6 (interquartile range [IQR], 6.9) months in the rezivertinib group and 6.2 (IQR, 4.0) months in the gefitinib group for patients with baseline CNS metastasis (*P* = 0.067), and the median time to the development of new CNS lesions was 12.4 (IQR, 8.3) months in the rezivertinib group and 8.3 (IQR, 9.6) months in the gefitinib group for patients without baseline CNS metastasis (*P* = 0.379; Table 3).

**Table 3.** Development of new CNS lesions by baseline CNS metastasis status

Development of new CNS lesions	With baseline CNS metastasis		Without baseline CNS metastasis	
	Rezivertinib (*n* = 81)	Gefitinib (*n* = 78)	Rezivertinib (*n* =103)	Gefitinib (*n* = 107)
Patients developed new CNS lesions, *n* (%)	15 (18.5)	14 (18.0)	5 (4.9)	14 (13.1)
Median time to develop new CNS lesions (IQR), months	9.6 (6.9)	6.2 (4.0)	12.4 (8.3)	8.3 (9.6)
*P* value	0.067		0.379	

CNS, central nervous system; IQR, interquartile range.

### Safety

In the cFAS, 81 (100.0%) patients who received rezivertinib and 75 (96.2%) patients who received gefitinib had at least 1 treatment-emergent adverse event (TEAE), of which 78 (96.3%) and 72 (92.3%) patients had treatment-related adverse events. Grade ≥3 TEAEs were 39 (48.1%) in the rezivertinib group and 29 (37.2%) in the gefitinib group, and grade ≥3 treatment-related adverse events were 20 (24.7%) in the rezivertinib group and 18 (23.1%) in the gefitinib group. Dose interruptions related to the study treatment were 8 (9.9%) in the rezivertinib group and 9 (11.5%) in the gefitinib group. Dose reductions related to the study treatment occurred in 12 (14.8%) in the rezivertinib group and 11 (14.1%) in the gefitinib group. Five (6.2%) patients in the rezivertinib group and 1 (1.3%) in the gefitinib group discontinued the study treatment owing to TEAEs. The overall safety result is available in Table S5.

## Discussion

In this study, rezivertinib showed superior CNS efficacy over gefitinib, reducing the risk of CNS progression or death by 42% and significantly prolonging CNS PFS. Rezivertinib also yielded higher CNS ORR and CNS DCR without new safety concerns. These findings were consistent with the REZOR study’s intention-to-treat population [13].

Previous studies established the promising CNS efficacy of rezivertinib as a second-line or later-line treatment in advanced NSCLC patients with *EGFR* T790M mutation [25–27] and as a first-line setting for *EGFR*-sensitizing mutations [13,36]. Specifically, our phase I study (NCT03386955) reported promising intracranial efficacy among 22 patients with target CNS lesions with an intracranial ORR of 50.0% (95% CI, 28.2% to 71.8%) and a DCR of 90.9% (95% CI, 70.8% to 98.9%), while the median intracranial DoR and time to progression were 11.2 months (95% CI, 2.8 to 12.4) and 13.9 months (95% CI, 6.9 months-not reached), respectively [25]. These findings were further supported by a subsequent phase IIb study (NCT03812809) involving 91 patients with CNS metastases, which reported a median CNS PFS of 16.6 months (95% CI, 11.1 months-NE) and a median CNS DoR of 15.2 months (95% CI, 8.3 months-NE). In the subgroup of 29 patients with at least 1 CNS target at baseline, the CNS ORR and CNS DCR were 69.0% (95% CI, 49.2% to 84.7%) and 100% (95% CI, 88.1% to 100%), respectively [26]. Moreover, in the phase IIa study (NCT03386955), promising CNS efficacy was observed in 12 (27.9%) treatment-naïve patients with baseline CNS metastasis, with a CNS ORR of 50.0% (95% CI, 21.1% to 78.9%) and a CNS DCR of 58.3% (95% CI, 27.7% to 84.8%). The BICR-assessed median PFS was 15.2 months (95% CI, 6.4 months-NE) in patients with baseline CNS metastasis and 22.0 months (95% CI, 13.8 months-NE) for those without baseline CNS metastasis (*P* = 0.399) [36]. Collectively, these consistent findings support rezivertinib as a promising first-line treatment option for patients with CNS metastatic NSCLC carrying *EGFR*-sensitizing mutations. In the cFAS population, rezivertinib achieved an approximately 2-fold higher CNS CR rate compared with gefitinib (25.9% versus 12.8%). Meanwhile, the CNS PFS rates at 6, 12, and 18 months were consistently higher with rezivertinib than with gefitinib in both the cFAS and cEFR populations. Notably, the difference was more pronounced in the cEFR population, suggesting that rezivertinib provides greater benefit in patients with larger baseline intracranial tumor volume/total sum of longest diameters. The cEFR results consistently suggested enhanced antitumor potency for rezivertinib, with patients achieving a deeper median target lesion reduction (−63.2% [range: −83.1% to −19.1%] versus −45.6% [range: −75.7% to 76.7%]; *P* = 0.157). This was reinforced by the finding that 75.0% (9/12) of patients in the rezivertinib group achieved ≥50% tumor shrinkage versus 38.5% (5/13) in the gefitinib group (*P* = 0.098), indicating a superior depth of CNS response. The difference of BOR with leptomeningeal lesions and CNS response between the 2 groups indicated that rezivertinib might have the potential to achieve long-term and effective control of leptomeningeal metastases when the simple size was larger.

Findings from the competing risk analysis underscored that, compared to gefitinib, rezivertinib revealed a lower estimated probability of CNS progression prior to non-CNS progression or death with rezivertinib among patients without baseline CNS metastasis. Meanwhile, rezivertinib showed a substantially lower probability of developing new CNS lesions among those without baseline CNS metastasis. Across patients with or without baseline CNS metastasis, rezivertinib demonstrated a longer median time to the occurrence of development with new CNS lesions. Taken together, these data suggested that rezivertinib could be associated with superior control of existing CNS disease and a lower risk and delayed occurrence of new CNS metastases compared to gefitinib.

In the FLAURA study, osimertinib as a first-line treatment reduced the risk of CNS progression or death by 52% compared to gefitinib or erlotinib. The CNS PFS was not reached (95% CI, 16.5 months-NE) with osimertinib versus 13.9 (95% CI, 8.3 months-NE) with gefitinib or erlotinib, with an HR of 0.48 (95% CI, 0.26 to 0.86, *P* = 0.014) [18]. In the AENEAS study, aumolertinib demonstrated significantly prolonged CNS PFS compared to gefitinib among *EGFR*-mutated treatment-naïve NSCLC patients. The CNS PFS was 29.0 months (95% CI, 12.3 months-NE) with aumolertinib versus 8.3 months (95% CI, 6.9 to 9.7) with gefitinib, with an HR of 0.319 (95% CI, 0.176 to 0.580, *P* < 0.0001) [20]. In the FURLONG study, the CNS PFS was 20.8 months (95% CI, 15.2 to 25.3) in the furmonertinib group and 9.8 months (95% CI, 7.2 to 18.0) in the gefitinib group (HR = 0.40, 95% CI, 0.23 to 0.71, *P* = 0.0011) [19]. In the limertinib phase III study, the CNS PFS was 20.7 months (95% CI, 5.4 months-NE) in the limertinib group and 7.1 months (95% CI, 5.5 to 11.0) in the gefitinib group (HR = 0.28, 95% CI, 0.10 to 0.82, *P* = 0.0136) [14]. Moreover, the reported CNS PFS rate of osimertinib, aumolertinib, furmonertinib, and rezivertinib was similar in these studies at 6, 12, and 18 months (FLAURA: 87%, 77%, and 58% [18]; AENEAS: 86%, 73%, and 60% [20]; FURLONG: 91%, 77%, and 63% [19]; REZOR: 89%, 72%, and 60% [Table 2]; respectively). All these third-generation EGFR TKIs showed superior CNS efficacy in patients with CNS metastasis over the first-generation EGFR TKIs as a first-line setting, which was consistent with the CNS efficacy of rezivertinib over gefitinib.

Studies have revealed that combination therapy could provide improved CNS PFS when compared with osimertinib monotherapy. In the FLAURA2 study, the median CNS PFS was 30.2 months (95% CI, 28.4 months-NE) for the osimertinib plus platinum-pemetrexed group and 27.6 months (95% CI, 22.1 months-NE) for the osimertinib monotherapy group (HR = 0.58, 95% CI, 0.33 to 1.01; *P* = 0.0548) [29]. In the MARIPOSA study, the median intracranial PFS was 25.4 months (95% CI, 20.1 to 29.5) for the amivantamab plus lazertinib group, while it was 22.2 months (95% CI, 18.4 to 26.9) for the osimertinib group (HR = 0.79, 95% CI, 0.61 to 1.02) [30]. Based on the favorable CNS efficacy of rezivertinib in the REZOR study, which was similar to that of osimertinib in the FLAURA study, it is plausible to consider that combination therapy with rezivertinib might further improve the CNS PFS when compared with monotherapy.

A key strength of this study is the mandatory inclusion of brain imaging via plain plus contrast-enhanced MRI during screening, with CNS metastasis used as a stratification factor during randomization. However, the study was limited by certain constraints. The absence of cerebrospinal fluid sampling presented a challenge in conclusively exploring leptomeningeal disease. Moreover, the limited sample size of the cEFR (*n* = 25) led to wide CIs and limited statistical power. Furthermore, caution should be taken when explaining the results to other ethnic groups, as only Chinese patients were enrolled in this study.

## Conclusions

In conclusion, rezivertinib demonstrated significant improvements in CNS outcomes compared with gefitinib as a first-line treatment for *EGFR*-mutated advanced NSCLC with baseline CNS metastasis, while maintaining a manageable safety profile.

## Ethical Approval

During the study, each participating hospital’s institutional review board or independent ethics committee approved the protocol and informed consent form (the approval number of the ethics committee of the leading hospital, National Cancer Center/Cancer Hospital, Chinese Academy of Medical Sciences, and Peking Union Medical College, was 19-039/1824; Table S1). Each participant in the study provided their informed consent in writing, voluntarily.

## Data Availability

Access to the data and materials that support the findings presented herein may be obtained from the corresponding author via a reasonable request.
