# Supplementary material for: Rezivertinib in EGFR-Mutated Non-Small Cell Lung Cancer Patients with Central Nervous System Metastasis: Central Nervous System Efficacy from the Phase III REZOR Study
Source: Cancer Commun (Lond). 2026 Mar 31;46:0018. doi: 10.34133/cancomm.0018 (PMC13036320; doi:10.34133/cancomm.0018)
Supplement: Supplementary 1 — Tables S1 to S5 [file cancomm.0018.f1.pdf]

Supplementary Materials for

Rezivertinib in EGFR-mutated non-small cell lung cancer patients with central nervous system metastasis: central nervous system efficacy from the phase 3 REZOR study

Sheng Yang[1], Yanqiu Zhao[2], Meili Sun[3], Minghong Bi[4], Bo Zhu[5], Zhaohong Chen[6], Huiqing Yu[7], Liangming Zhang[8], Lin Wu[9], Rui Zhou[10], Wenxiu Yao[11], Xingya Li[12], Zhigang Han[13], Ke Wang[14], Lijun Wang[15], Meiling Wen[16], Yanzhen Guo[17], Yingcheng Lin[18], Shenghua Sun[19], Shuliang Guo[20], Tienan Yi[21], Wenhua Zhao[22], Zhuang Yu[23], Jianwen Qin[24], Yueyin Pan[25], Zhiyong He[26], Feng Ye[27], Huaqiu Shi[28], Jian Fang[29], Rui Ma[30], Hong Lu[31], Hua Zhang[32], Jianhua Shi[33], Jinghua Gao[34], Jiuwei Cui[35], Manxiang Li[36], Shanyong Yi[37], Shundong Cang[38], Yongqian Shu[39], Don Zhang[40], Jirong Peng[40], Feng Gao[41], Tingting Wang[41], Anqi Zhou[41], Yuankai Shi[1,]*

[1] Department of Medical Oncology, Beijing Key Laboratory of Key Technologies for Early Clinical Trial Evaluation of Innovative Drugs for Major Diseases, National Cancer Center/National Clinical Research Center for Cancer/Cancer Hospital, Chinese Academy of Medical Sciences & Peking Union Medical College, Beijing, P. R. China

[2] Respiratory Department of Internal Medicine, The Affiliated Cancer Hospital of Zhengzhou University & Henan Cancer Hospital, Zhengzhou, Henan, P. R. China

[3] Department of Oncology, Central Hospital Affiliated to Shandong First Medical University, Jinan, Shandong, P. R. China

[4] Department of Medical Oncology, The First Affiliated Hospital of Bengbu Medical University, Bengbu, Anhui, P. R. China

[5] Department of Oncology, Institute of Cancer, Xinqiao Hospital, Third Military Medical University, Chongqing, P. R. China

[6] Department of Oncology, People's Hospital of Deyang City, Deyang, Sichuan, P. R. China

[7] Department of Geriatric Oncology, Chongqing University Cancer Hospital, Chongqing, P. R. China

[8] Department of Medical Oncology, Yantai Yuhuangding Hospital, Yantai, Shandong, P. R. China

[9] Department of Thoracic Medical Oncology, Hunan Cancer Hospital/The Affiliated Cancer Hospital of Xiangya School of Medicine, Central South University, Changsha, Hunan, P. R. China

[10] Department of Respiratory and Critical Care Medicine, The Second Xiangya Hospital of Central South University, Changsha, Hunan, P. R. China

[11] Department of Medical Oncology, Sichuan Cancer Hospital/Cancer Hospital Affiliated to University of Electronic Science and Technology of China, Chengdu, Sichuan, P. R. China

[12] Department of Oncology, The First Affiliated Hospital of Zhengzhou University, Zhengzhou, Henan, P. R. China

[13] First Department of Pulmonary Medicine, Affiliated Cancer Hospital of Xinjiang Medical University, Urumqi, Xinjiang, P. R. China

[14] Department of Respiratory and Critical Care Medicine, West China Hospital of Sichuan University, Chengdu, Sichuan, P. R. China

[15] Cancer Center, The Second Affiliated Hospital of Xingtai Medical College, Xingtai, Hebei, P. R. China

[16] Department of Medical Oncology, The First Affiliated Hospital of University of South China, Hengyang, Hunan, P. R. China

[17] Department of Medical Oncology, The First Affiliated Hospital of Henan University of Science & Technology, Luoyang, Henan, P. R. China

[18] Department of Medical Oncology, Cancer Hospital of Shantou University Medical College, Shantou, Guangdong, P. R. China

[19] Department of Pulmonary and Critical Care Medicine, Third Xiangya Hospital of Central South University, Changsha, Hunan, P. R. China

[20] Department of Respiratory and Critical Care Medicine, The First Affiliated Hospital of Chongqing Medical University, Chongqing, P. R. China

[21] Department of Medical Oncology, Xiangyang Central Hospital, Xiangyang, Hunan, P. R. China

[22] Department of Internal Medicine for Lung Cancer, Guangxi Medical University Cancer Hospital, Nanning, Guangxi, P. R. China

[23] Department of Medical Oncology, The Affiliated Hospital of Qingdao University, Qingdao, Shandong, P. R. China

[24] Department of Respiratory and Critical Medicine, Chest Hospital, Tianjin University, Tianjin, P. R. China

[25] Department of Oncology, The First Affiliated Hospital of University of Science and Technology of China, Division of Life Sciences and Medicine, University of Science and Technology of China, Hefei, Anhui, P. R. China

[26] Department of Medical Oncology, Clinical Oncology School of Fujian Medical University, Fujian Cancer Hospital, National Health Commission (NHC) Key Laboratory of Cancer Metabolism, Fuzhou, Fujian, P. R. China

[27] Department of Medical Oncology, Xiamen Key Laboratory of Antitumor Drug Transformation Research, The First Affiliated Hospital of Xiamen University, School of Medicine, Xiamen University, Xiamen, Fujian, P. R. China

[28] Department of Medical Oncology, First Affiliated Hospital of Gannan Medical University, Ganzhou, Jiangxi, P. R. China

[29] Department of Thoracic Oncology, Beijing Cancer Hospital, Beijing, P. R. China

[30] Department of Thoracic Oncology, Liaoning Cancer Hospital & Institute, Shenyang, Liaoning, P. R. China

[31] Department of Oncology, Huaihe Hospital of Henan University, Kaifeng, Henan, P. R. China

[32] Department of Medical Oncology, The First Affiliated Hospital of Xinjiang Medical University, Urumqi, Xinjiang, P. R. China

[33] Department of Medical Oncology, Linyi Cancer Hospital, Linyi, Shandong, P. R. China

[34] Department of Medical Oncology, Cangzhou Central Hospital, Cangzhou, Hebei, P. R. China

[35] Oncology Center, Oncology Department, First Hospital of Jilin University, Changchun, Jilin, P. R. China

[36] Department of Respiratory and Critical Care Medicine, The First Affiliated Hospital of Xi'an Jiaotong University, Xi'an, Shaanxi, P. R. China

[37] Department of Medical Oncology, Zhengzhou Central Hospital Affiliated to Zhengzhou University, Zhengzhou, P. R. China

[38] Department of Oncology, Henan Provincial People's Hospital, Zhengzhou, Henan, P. R. China

[39] Department of Oncology, The First Affiliated Hospital of Nanjing Medical University (Jiangsu Province Hospital), Nanjing, Jiangsu, P. R. China

[40] Department of Drug Discovery, Beta Pharma Inc., Princeton, New Jersey, USA

[41] Department of Clinical Development, Beta Pharma (Shanghai) Co., Ltd., Shanghai, P. R. China

***Corresponding author**

Yuankai Shi

Department of Medical Oncology, Beijing Key Laboratory of Key Technologies for Early Clinical Trial Evaluation of Innovative

Drugs for Major Diseases, National Cancer Center/National Clinical Research Center for Cancer/Cancer Hospital, Chinese Academy of Medical Sciences & Peking Union Medical College, Beijing 100021, P. R. China, Email: syuankai@cicams.ac.cn

Supplementary Tables

Table S1. Ethics approval information for 50 participating institutions

PI	No. of Enrolled Patients	Institution Name	Ethics Approval ID
Yuankai Shi/Sheng Yang	5	Department of Medical Oncology, Beijing Key Laboratory of Key Technologies for Early Clinical Trial Evaluation of Innovative Drugs for Major Diseases, National Cancer Center/National Clinical Research Center for Cancer/Cancer Hospital, Chinese Academy of Medical Sciences & Peking Union Medical College, Beijing, P. R. China	19-039/1824
Jian Fang	2	Department of Thoracic Oncology, Beijing Cancer Hospital, Beijing, P. R. China	2019YW163
Yongqian Shu	1	Department of Oncology, The First Affiliated Hospital of Nanjing Medical University (Jiangsu Province Hospital), Nanjing, P. R. China	2019-MD-199
Mingyan Jiang	0	Department of Respiratory and Critical Care Medicine, Xiangtan Central Hospital, Hunan, P. R. China	2021-05-13-5
Bo Zhu	7	Department of Oncology, Institute of Cancer, Xinqiao Hospital, Third Military Medical University, Chongqing, P. R. China	2019-药第 013-01
Xingya Li	6	Department of Oncology, The First Affiliated Hospital of Zhengzhou University, Zhengzhou, P. R. China	药-2019-049
Hongbo Ji	0	Department of Medical Oncology in Section One, Chifeng Municipal Hospital, Inner Mongolia, P. R. China	2021-LLSCZN-062-21
Meiling Wen	5	Department of Medical Oncology, The First Affiliated Hospital of University of South China, Hengyang, P. R. China	[2021]药伦审字(25-01)号
Minghong Bi	8	Department of Medical Oncology, The First Affiliated Hospital of Bengbu Medical University, Bengbu, P. R. China	[2019]040X01 号
Huiqing Yu	6	Department of Geriatric Oncology, Chongqing University Cancer Hospital, Chongqing, P. R. China	CZLL2019113
Haitao Wang	0	Department of Medical Oncology, The Second Hospital of Tianjin Medical University, Tianjin, P. R. China	临审[2021]第（016）号
Yanqiu Zhao	11	Respiratory Department of Internal Medicine, The Affiliated Cancer Hospital of Zhengzhou University & Henan Cancer Hospital, Zhengzhou, P. R. China	2019191
Jinghua Gao	1	Department of Medical Oncology, Cangzhou Central Hospital, Cangzhou, P. R. China	2021-029-01
Jianhua Shi	1	Department of Medical Oncology, Linyi Cancer Hospital, Linyi, P. R. China	NA
Liangming Zhang	6	Department of Medical Oncology, Yantai Yuhuangding Hospital, Yantai, P. R. China	【2019】16 号
Jianwen Qin	3	Respiratory and Critical Care Department, Tianjin Chest Hospital, Tianjin, P. R. China	2021-014-01
Yun Fan	0	Department of Thoracic Medical Oncology, Zhejiang Cancer Hospital, Zhejiang, P. R. China	IRB-[2021]968 号
Tienan Yi	4	Department of Medical Oncology, Xiangyang Central Hospital, Xiangyang, P. R. China	XYSZXYY-LL-PJ-2019-007
Manxiang Li	1	Department of Respiratory and Critical Care Medicine, The First Affiliated Hospital of Xi'an Jiaotong University, Xi'an, P. R. China	2019 伦审药临字第(37)号
Shenghua Sun	4	Department of Respiratory Medicine, Third Xiangya Hospital of Central South University, Changsha, P. R. China	19048、21060、快 19090
Wenxiu Yao	6	Department of Medical Oncology, Sichuan Cancer Hospital/Cancer Hospital Affiliated to University of Electronic Science and Technology of China, Chengdu, P. R. China	SCCHEC-01A-2021-058

Ke Wang	5	Department of Respiratory and Critical Care Medicine, West China Hospital of Sichuan University, Chengdu, P. R. China	2019 年临床试验（西药）审（62）号
Hua Zhang	1	Department of Medical Oncology, The First Affiliated Hospital of Xinjiang Medical University, Urumqi, P. R. China	D190325-02
Lijun Wang	5	Cancer Center, The Second Affiliated Hospital of Xingtai Medical College, Xingtai, P. R. China	XYEYCTEC-HS-019
Lin Wu	6	Department of Thoracic Medical Oncology, Hunan Cancer Hospital/The Affiliated Cancer Hospital of Xiangya School of Medicine, Central South University, Changsha, P. R. China	2019 药审[115]号
Zhiyong He	*3*	Thoracic Medical Oncology, Fujian Cancer Hospital, Fuzhou, P. R. China	2019-055-01
Zhuang Yu	4	Department of Medical Oncology, The Affiliated Hospital of Qingdao University, Qingdao, P. R. China	2019-038-01
Yanzhen Guo	5	Department of Medical Oncology, The First Affiliated Hospital of Henan University of Science & Technology, Luoyang, P. R. China	2019-0037
Diansheng Zhong	0	Department of Medical Oncology, Tianjin Medical University General Hospital, Tianjin, P. R. China	IRB2019-060-01
Shanyong Yi	1	Department of Medical Oncology, Zhengzhou Central Hospital Affiliated to Zhengzhou University, Zhengzhou, P. R. China	2019-031-01
Wenhua Zhao	4	Department of Internal Medicine for Lung Cancer, Guangxi Medical University Cancer Hospital, Nanning, P. R. China	CS2020(12)、KS2021（39）
Yan Zhang	0	Department of Medical Oncology, Shijiazhuang People's Hospital, Hebei, P. R. China	SJZSRMYY-2021-10-01
Yingcheng Lin	5	Department of Medical Oncology, Cancer Hospital of Shantou University Medical College, Shantou, P. R. China	2019013
MingleiYang	0	Department of Thoracic Surgery, Ningbo No.2 Hospital, Zhejiang, P. R. China	PJ-NBEY-2019-005-01
Meili Sun	8	Department of Oncology, Central Hospital Affiliated to Shandong First Medical University, Jinan, P. R. China	2019-032-01
Zhigang Han	6	Pulmonary Cancer Medicine, Affiliated Tumor Hospital of Xinjiang Medical University, Urumqi, P. R. China	[2019]伦审字(025)
Sheng Hu	0	Department of Thoracic Oncology, Hubei Cancer Hospital, Hubei, P. R. China	【2021】第 62 号
Rui Ma	2	Department of Thoracic Oncology, Liaoning Cancer Hospital & Institute, Shenyang, P. R. China	20190745-1
Jiuwei Cui	1	Oncology Center, Oncology Department, First Hospital of Jilin University, Changchun, P. R. China	19Y067-001
Rui Zhou	6	Department of Respiratory Medicine, The Second Xiangya Hospital of Central South University, Changsha, P. R. China	(2019)伦审[药]第(035)号
Shundong Cang	1	Department of Oncology, Henan Provincial People's Hospital, Zhengzhou, P. R. China	2019-028-01
Shuliang Guo	4	Department of Respiratory Medicine, The First Affiliated Hospital of Chongqing Medical University, Chongqing, P. R. China	2019 年伦审（CY20193101）
Zhaohong Chen	7	Department of Oncology, People's Hospital of Deyang City, Deyang, P. R. China	2019-01-003-H01
Feng Ye	2	Department of Medical Oncology, Xiamen Key Laboratory of Antitumor Drug Transformation Research, The First Affiliated Hospital of Xiamen University, School of Medicine, Xiamen University, Xiamen, P. R. China	XMYY-2019Y055-01
Wen Li	0	Department of Respiratory and Critical Care Medicine, The Second Affiliated Hospital Zhejiang University School of Medicine, Zhejiang, P. R. China	(2019)伦审药第(391)号
Hong Lu	1	Department of Oncology, Huaihe Hospital of Henan University, Kaifeng, P. R. China	2019-01-031-H01
Yueyin Pan	3	Department of Oncology, The First Affiliated Hospital of University of Science and Technology of China, Division of Life Sciences and	2019 伦审第 178 号

		Medicine, University of Science and Technology of China, Hefei, P. R. China	
Huaqiu Shi	2	Department of Medical Oncology, First Affiliated Hospital of Gannan Medical University, Ganzhou, P. R. China	(2019)赣医伦审 038号
Zheng Liu	0	Department of Oncology, Handan Central Hospital, Hebei, P. R. China	（2019）伦审药第（010）号
Xiangdong Zhou	0	Department of Respiratory and Critical Care Medicine, The First Affiliated Hospital of Army Medical University of Chinese People's Liberation Army, Chongqing, P. R. China	(BA)YW202173

Table S2. CNS response of patients with baseline leptomeningeal lesions(cFAS) [a]

Treatment arm	CNS BOR	CNS PFS, months	CNS PFS event or censored
Rezivertinib	PR	19.3	Censored (no progression or death)
Rezivertinib	PR	20.7	Event (progression)
Rezivertinib	Non-CR/non-PD	15.4	Event (death)
Rezivertinib	Non-CR/non-PD	22.1	Censored (new anti-cancer therapy)
Rezivertinib	Non-CR/non-PD	1.4	Censored (new anti-cancer therapy)
Gefitinib	PR	6.1	Event (death)
Gefitinib	Non-CR/non-PD	19.3	Censored (no progression or death)
Gefitinib	Non-CR/non-PD	13.8	Censored (new anti-cancer therapy)
Gefitinib	PR	19.4	Censored (no progression or death)
Gefitinib	Non-CR/non-PD	10.6	Event (death)

[a]Two patients from each group were from the cEFR population. For patients listed above, leptomeningeal lesions were assessed as non-target lesions; and the responses for leptomeningeal lesions were all evaluated as non-CR/non-PD. Abbreviations: BOR, best overall response; cFAS, CNS full analysis set; CNS, central nervous system; CR, complete response; FAS, full analysis set; PR, partial response; PD, progressive disease; PFS, progression-free survival.

Table S3. Best percentage change in CNS target lesions from baseline in cEFR assessed by BICR

**Best percentage change in target lesions from baseline**	**Rezivertinib (*n* = 12)**	**Gefitinib (*n* = 13)[a]**
Median (range)[b], %	-63.2 (-83.1, -19.1)	-45.6% (-75.7, 76.7)
≥0% increase, *n* (%)	0 (0.0)	1 (7.7)
≥0% to <30% reduction, *n* (%)	2 (16.7)	1 (7.7)
≥30% to <50% reduction, *n* (%)	1 (8.3)	5 (38.5)
≥50% reduction[c], *n* (%)	9 (75.0)	5 (38.5)

[a] One patient had no post-baseline CNS response assessment results.

[b] The *P* value between the two groups was 0.157

[c] The *P* value between the two groups was 0.098.

Abbreviations: BICR, blinded independent central review; cEFR, CNS evaluable for response set; CNS, central nervous system.

Table S4. Cumulative incidence of CNS progression in patients with and without CNS metastasis at baseline.

Variables	With CNS metastasis at baseline		Without CNS metastasis at baseline	
	**Rezivertinib (*n* = 81)**	**Gefitinib (*n* = 78)**	**Rezivertinib (*n* =103)**	**Gefitinib (*n* = 107)**
Censored, *n* (%)	35 (43.2)	24 (30.8)	52 (50.5)	31 (29.0)
Event, *n* (%)				
CNS progression	15 (18.5)	14 (17.9)	5 (4.9)	14 (13.1)
Progression	24 (29.6)	35 (44.9)	42 (40.8)	56 (52.3)
Death	7 (8.6)	5 (6.4)	4 (3.9)	6 (5.6)
Cumulative incidence rate (95% CI), %				
At 6 months	4.1 (1.1-10.5)	10.2 (4.4-18.9)	1.0 (0.1-4.8)	6.0 (2.5-11.9)
At 12 months	14.2 (7.2-23.5)	18.6 (10.1-29.1)	2.2 (0.4-6.9)	10.5 (5.3-17.7)

Abbreviations: CNS, central nervous system; CI, confidence interval.

Table S5. Safety summary of the cFAS

Adverse events	Rezivertinib (n = 81), n (%)	Gefitinib (n = 78) n (%)	Total (n = 159), n (%)
Number of patients with at least one TEAE	81 (100.0)	75 (96.2)	156 (98.1)
Grade ≥3	39 (48.1)	29 (37.2)	68 (42.8)
Grade 3	30 (37.0)	23 (29.5)	53 (33.3)
Grade 4	4 (4.9)	4 (5.1)	8 (5.0)
Grade 5	5 (6.2)	2 (2.6)	7 (4.4)
Number of patients with at least one TRAE	78 (96.3)	72 (92.3)	150 (94.3)
Grade ≥3	20 (24.7)	18 (23.1)	38 (23.9)
Grade 3	19 (23.5)	14 (17.9)	33 (20.8)
Grade 4	1 (1.2)	4 (5.1)	5 (3.1)
Grade 5	0 (0.0)	0 (0.0)	0 (0.0)
TEAE leading to death	5 (6.2)	2 (2.6)	7 (4.4)
TRAE leading to death	0 (0.0)	0 (0.0)	0 (0.0)
TEAE leading to treatment termination	8 (9.9)	1 (1.3)	9 (5.7)
TRAE leading to treatment termination	5 (6.2)	1 (1.3)	6 (3.8)
TEAE leading to dose adjustment	23 (28.4)	19 (24.4)	42 (26.4)
TEAE leading to dose interruption	18 (22.2)	12 (15.4)	30 (18.9)
TEAE leading to dose reduction	12 (14.8)	11 (14.1)	23 (14.5)
TRAE leading to dose adjustment	16 (19.8)	17 (21.8)	33 (20.8)
TRAE leading to dose interruption	8 (9.9)	9 (11.5)	17 (10.7)
TRAE leading to dose reduction	12 (14.8)	11 (14.1)	23 (14.5)
Serious TEAEs	21 (25.9)	20 (25.6)	41 (25.8)
Serious TRAEs	7 (8.6)	8 (10.3)	15 (9.4)
Serious TEAEs leading to discontinuation of study drug	5 (6.2)	1 (1.3)	6 (3.8)

Note: Safety was determined by the investigator as per CTCAE Version 4.03. Abbreviations: CTCAE, Common Terminology Criteria for Adverse Events; FAS, full analysis set; cFAS, CNS full analysis set; CNS, central nervous system; TEAE, treatment-emergent adverse event; TRAE, treatment-related adverse event.
